# Susceptibility scoring in family-based association testing

**DOI:** 10.1186/1471-2156-4-S1-S49

**Published:** 2003-12-31

**Authors:** Laila M Poisson, Benjamin A Rybicki, Steven W Coon, Jill S Barnholtz-Sloan, Gary A Chase

**Affiliations:** 1 Department of Biostatistics and Research Epidemiology, Henry Ford Health Sciences Center, Detroit, Michigan, USA; 2 Karmanos Cancer Institute, Wayne State University, Detroit, Michigan, USA; 3 Department of Health Evaluation Sciences, Penn State Hershey Medical Center, Hershey, Pennsylvania, USA

## Abstract

**Background:**

Family-based association testing is an important part of genetic epidemiology. Tests are available to include multiple siblings, unaffected offspring, and to adjust for environmental covariates. We explore a susceptibility residual method of adjustment for covariates.

**Results:**

Through simulation, we show that environmental adjustments that down-weight persons who are "destined" to be affected decrease the power to detect genetic association. We used the residual adjusted method on the Framingham Heart Study offspring data, provided for Genetic Analysis Workshop 13, and got mixed results.

**Conclusion:**

When the genetic effect and environmental effects are independent, a susceptibility residual method of adjustment for environmental covariates reduces the power of the association test. Further study is necessary to determine if residual adjustment is appropriate in more complex disease models.

## Background

Family-based association tests, such as Spielman's transmission disequilibrium test (TDT) [1] rely on affection status and marker phenotypes for determination of within-family association of disease and allele. The original version of the TDT, still frequently used in association studies of complex phenotypes, scores only affected offspring. The modified family-based association test (FBAT) method by Horvath et al. [2,3] can use a covariate-adjusted phenotypic value allowing unaffected offspring to contribute to the test statistic [4,5]. The covariate-adjusted phenotypic value, **T = Y - μ**, adjusts **Y**, a dichotomous indicator of affection status, by μ, a covariate value based on a dichotomous trait, measured trait value (e.g., glucose), or age-dependent phenotypic value.

To expand this covariate adjustment to a more general setting, we propose the use of a susceptibility score. For instance, we model affection status on the covariates of interest using logistic regression and then use the susceptibility residual as a measure of deviance from the predicted outcome. Genotypes are excluded from the regression because we are not adjusting for genetic effects. Our logistic model differs only slightly from the suggested calculation of **T**, such that **T = Y - p**, where **p **is the estimated predicted probability from the logistic model. In this context, we call **T **the susceptibility residual.

A concern with the covariate adjustment method in FBAT is that persons with low deviance from the predicted outcome are heavily down-weighted. The assumption is that persons with low deviance were susceptible to affection regardless of genetic makeup and therefore should not contribute fully to the association test. In the extreme case in which the affection status is predicted perfectly by the covariate in FBAT, that individual contributes no information to the result. Through simulation we explore the power and type I error rates of the adjusted Horvath test compared to the unadjusted version of the same test. We vary the influence of the genetic component and the non-genetic (covariate) component from no influence to an increased odds-ratio of 4.95. This comparison of methods allows us to investigate the advantages and disadvantages of covariate adjustment. Briefly stated, we found that for those cases in which a moderately strong genetic influence is determinative, adjustment for environmental cofactors can reduce the power to detect association between the disease and the disease allele. We apply the susceptibility residual adjustment to the Framingham Heart Study offspring data, available for the Genetic Analysis Workshop 13 (GAW13), to observe the effects of adjustment on nonsimulated data.

## Methods

### Data simulation and simulated disease onset

In each replicate 100 families were generated with two parents and 2 to 10 offspring, according to a truncated Poisson distribution (λ = 4), such that each family contained at least one affected offspring. Parental genotypes were determined by random selection assuming Hardy-Weinberg equilibrium and random inheritance was used for determining offspring genotypes. For these simulations, a bi-allelic marker was considered such that the disease allele has a population frequency of 0.15. Offspring exposure to the covariate was randomly determined using a population exposure rate of 0.20. Disease status was determined by the following logistic model:



where α ≈ -2.9, such that Pr(*disease*|*x*_1 _= *x*_2 _= 0) = 0.05 and β_1_, β_2 _are the regression coefficients corresponding to the indicator variables x_1 _and x_2 _for raised genetic and environmental risk, respectively. The β values were varied at intervals of 0.2 from 0.0, no risk, to 1.6, increased odds of 4.95, thus creating nine levels of the genetic effect and nine levels of the covariate effect. The probability of disease calculated by the above model was compared to a random number selected from a uniform [0,1] distribution. If the probability of disease onset is greater than the random number, then the individual in question is labeled as affected.

### Generation of residuals

Residuals were created from a logistic model with affection status as the outcome and a single dichotomous covariate indicating exposure. Since each family has at least two offspring, a robust sampling method was used to avoid issues of correlated data within families. The logistic model was fitted 100 times on randomly selected subsets of the offspring, such that each family was represented by a single offspring. Average parameter values from the 100 cycles were used to generate residuals for each offspring.

### FBAT

A bi-allelic marker with dominant disease inheritance was simulated and thus the dominant model and bi-allelic test mode were used in FBAT. We ran FBAT twice on each simulated data set to observe the results from both the covariate adjusted and unadjusted affection status tests. Significant results were determined by a *p*-value less than or equal to 0.05 for the disease allele. Power was calculated as the frequency of significant results per 1000 cycles. The type I error rate of the model was calculated as the frequency of significant results per 1000 cycles when no genetic effect was modeled.

### Framingham Heart Study offspring data

To demonstrate our assumptions from the simulated data in a real world setting, we applied the FBAT procedure to the Framingham Heart Study offspring data, available for GAW13, using offspring from Cohort 2 and their parents if available. The disease status of interest was hypertension, determined by systolic blood pressure measurement over 140 for three consecutive exams or by the administration of anti-hypertensive drugs. We used regular smoking as the environmental covariate, determined by a self-report of smoking at least 20 cigarettes (1 pack) per day for at least one of the five exams.

The genotypes of interest were determined using a genome scan with FBAT on a random selection of nuclear families. We selected markers GATA48G07A (10q25.3) and GGAA5D10 (10q26.13) on chromosome 10, and GGAA7D11 (17q11.2) on chromosome 17 because each showed a significant association to hypertension using FBAT. Also, literature suggests association for neighboring regions on chromosome 10 [6,7] and chromosome 17 [8-10]. As negative controls, we selected markers GATA88F09 (10p15.3), at least 130 cM away from the region of interest on chromosome 10, and 217YD10 (17q25.3), approximately 70 cM away from the region of interest on chromosome 17. Neither negative control marker had shown significant association with hypertension in the FBAT genome scan and thus we used them to explore false positive rates in the nonsimulated data.

To match the methods of the simulation, FBAT was run using a dominant model of inheritance and the bi-allelic testing mode. Significance of an allele was determined by *p *< 0.05. The marker was tested on 100 random selections of nuclear families from the full pedigree data. Thus correlation between families within a pedigree was avoided. The program default minimum of 10 informative families for testing was maintained in these FBAT runs.

## Results

### Power and type I error

**Figure 1:**
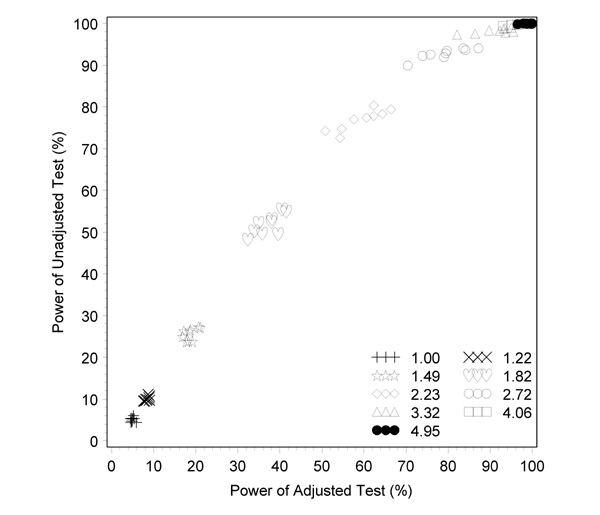
**Power **Plot of power for the adjusted test versus the unadjusted test at varying genetic risk levels. The legend lists the odds of disease, given inheritance of the disease allele, for each risk cluster. Each of the nine covariate risk levels is represented within a single genetic risk cluster. There is a tendency for higher environmental risk to decrease the power within a genetic risk cluster (not shown).

**Figure 2:**
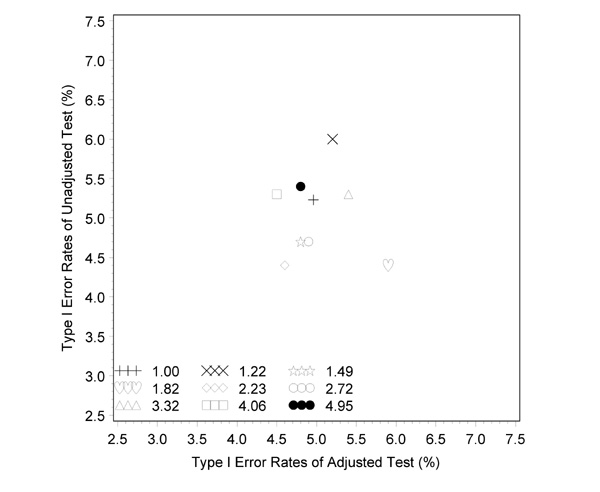
**Type I error rates **Plot of type I error rates for the adjusted test versus the unadjusted test at varying covariate risk levels. The legend lists the odds of disease, given exposure to the covariate, at each point. The type I error rates are not significantly different from 5% for either the adjusted or unadjusted test (*p *> 0.5). Likewise, the error rates are not significantly different between tests (*p *= 0.71).

The power of the FBAT procedure was determined for each parameter set by testing 1000 simulated data sets, 100 families each, and recording the frequency of significant results (*p *< 0.05 for the disease allele). Figure 1 plots the power of the test for the adjusted versus the unadjusted method at each level of risk combinations. Formal testing of the two methods, at each of the eight non-zero levels of genetic effect, shows that the unadjusted method has consistently higher power than the adjusted method (*p *< 0.05, with Benjamini-Hochberg correction for multiple comparisons). The type I error rate of the model was determined at varying covariate effect levels, using no genetic effect. Figure 2 plots the type I error rates of the test for the adjusted versus unadjusted methods at each of the nine covariate levels. The type I error rates did not differ significantly between the two test methods when compared using a Wilcoxon signed rank test on the pair-wise differences (*p *= 0.71). Thus we find no change in type I error rates from the covariate adjustment.

### Framingham Heart Study offspring data

The comparison of the test methods, unadjusted versus adjusted, on the Framingham Heart Study offspring data shows mixed results. Tables 1,2,3 list the proportion of tests that produced a significant result for each allele, at markers GATA48G07A (c10), GGAA5D10 (c10), and GGAA7D11 (c17), respectively. One hundred random selections of nuclear families were tested and a minimum of 10 informative families at an allele were required for testing. Alleles for which no tests were done are excluded from Tables 1,2,3. Note the increase in the number of informative families, per allele, for the adjusted method compared to the unadjusted method. The covariate-adjusted method provides more information to the phenotype and thus most alleles have twice as many informative families compared to the unadjusted method.

In Table 1, marker GATA48G07A, allele 350 shows 100% significance and is under-transmitted for both testing methods. However, the unadjusted test was only run on 47 of the 100 random samples due to a lack of informative families (median *N *= 9). The increased information in the adjusted test allowed it to run on all 100 random samples (median *N *= 23). Allele 362 is over-transmitted and shows 65% significance in the unadjusted method and only 42% significance in the adjusted method, suggesting reduced power in the adjusted method.

In Table 2, marker GGAA5D10, we see increased transmission for alleles 93 and 109. The unadjusted test had 66% significant tests for allele 93 versus only 8% significance with the adjusted test. Likewise, the unadjusted test had 91% significant tests for allele 109 versus only 75% significance with the adjusted test. However, the adjusted test detected significantly decreased transmission in allele 105 in 22% of the 100 random samples versus only 3% in the unadjusted test.

In Table 3, marker GGAA7D11, we see increased transmission in allele 286 with 35% significance using the unadjusted test versus only 6% significance in the adjusted test. Allele 274 showed decreased transmission with 87% significance using the unadjusted FBAT test compared to 89% significance using the adjusted test. Significantly increased transmission was shown for allele 262 in 46% of the 100 random samples using the adjusted test. Increased transmission at allele 262 was not detected by the unadjusted test.

Results for the negative control markers show low false-positive rates for both methods, data not shown. At marker GATA88F09, the negative control on chromosome 10, the unadjusted test showed no significant results (0% false positive) and the adjusted test showed a low number of significant results (0–7% false positive, mean < 2.5%) on the five alleles tested. At marker 217YD10, the negative control on chromosome 17, the unadjusted test showed no significant results (0% false positive) for all but one of the seven alleles tested (30% false positive). The adjusted test at marker 217YD10 showed a low number of significant results (0–8% false positive, mean < 1%) for the 11 alleles tested.

## Discussion

In simulated data the covariate adjustment procedures in FBAT demonstrated the expected loss in power attributable to the "discounting" of information for affected persons who had a high prior environmental risk of disease. This phenomenon of "discounting" has the paradoxical attribute that more definitive information about the environmental contributors to a trait may actually cause more difficulty in detecting a genetic signal.

Interpretation of the comparison between the adjusted and unadjusted versions of the FBAT test when applied to real data is difficult primarily because the suggested loss of power was not uniform across marker loci. In particular, at the three markers a total of eight alleles were detected by one or both of the tests, of which four were detected more frequently with the unadjusted test and three were detected more frequently with the adjusted test. All four of the alleles detected more frequently by the unadjusted test had increased transmission. Yet only two of the three alleles detected more frequently by the adjusted test were under-transmitted and allele 350 on marker GATA48G07A, detected in 100% of the tests for both methods, is over-transmitted. Also, no pattern arises between allele frequency and the frequency of detection by either test method, unadjusted or adjusted.

The negative controls suggest that the false-positive rates for both tests are low; however, the spurious 30% false-positive rate presumed for one allele on marker 217YD10 (chromosome 10) for the unadjusted test lends doubt to the results for alleles showing significant association at low frequencies. In particular the significance of allele 286 on marker GGAA7D11 (Table 3) may be spurious. Yet, false-positive rates were low (0–8% false positive) for all alleles when the adjusted test was used so it is not appropriate to disregard all low frequency significant results. Thus false-positive rates cannot explain the discrepant results between the two tests.

The mixed results, regarding power loss in the susceptibility residual adjusted test, from the three markers of interest could reflect variation in the extent of genetic determination of the trait or possibly interaction between genetic and environmental factors that is not specified in our model. Further simulations, accounting for variable influence of different alleles at a marker and gene × environment interaction, would be needed to explain this set of results more satisfactorily.

## Conclusions

When the genetic effect and environmental effects are independent, a susceptibility residual method of adjustment for environmental covariates reduces the power of the association test. Results of the method comparisons in the Framingham data are not conclusive and further study is necessary to determine if susceptibility residual adjustment is appropriate in more complex disease models.

Our results should not be taken as indication that the FBAT adjustment should be avoided. Further simulations, involving correlation between genetic and covariate influences, are being explored in an effort to find situations in which the susceptibility residual is beneficial for the adjustment of covariate effects. Likewise, the reader should note that we have explored only one method of covariate adjustment for family-based association testing. We hope that our efforts in this matter will encourage others to evaluate covariate adjustment measures, since we believe that accounting for nongenetic influences is important in modeling complex diseases.

## Figures and Tables

**Table 1:** Results for marker GATA48G07A (chromosome 10) Results from FBAT analysis of the Framingham Heart Study offspring data using the unadjusted test for association between marker GATA48G07A (chromosome 10) and high blood pressure and the adjusted test using regular smoking (>1 pack/day) as a covariate.

Allele		Unadjusted Test			Adjusted Test		
		
Name^A^	Frequency	No. Tests	% Significance	Median^B ^*N*	No. Tests	% Significance	Median^B ^*N*
346	0.028	0	--	6	100	0	13
350	0.063	47	100	9	100	100	23
354	0.208	100	0	22	100	0	52
358	0.309	100	0	29	100	0	61
362	0.149	100	65	21	100	42	54
366	0.097	100	0	15	100	1	35
370	0.041	0	--	4	100	0	16
374	0.050	76	0	10	100	0	23
378	0.041	0	--	7	100	0	15

^A^Alleles 330, 338, 342, 382, and 386 did not have any informative tests. ^B^*N *= median number of informative families.

**Table 2:** Results for marker GGAA5D10 (chromosome 10) Results from FBAT analysis of the Framingham Heart Study offspring data using the unadjusted test for association between marker GGAA5D10 (chromosome 10) and high blood pressure and the adjusted test using regular smoking (>1 pack/day) as a covariate.

Allele		Unadjusted Test			Adjusted Test		
		
Name^A^	Frequency	No. Tests	% Significance	Median^B ^*N*	No. Tests	% Significance	Median^B ^*N*
93	0.031	100	66	17	100	8	28
101	0.081	100	2	31	100	0	66
105	0.179	100	3	52	100	22	107
109	0.366	100	91	55	100	75	138
113	0.118	100	0	36	100	0	80
117	0.102	100	0	34	100	0	70
121	0.083	100	0	25	100	0	62
125	0.026	100	0	11	100	0	26
129	0.010	0	--	5	7	14	8

^A^Alleles 97 and 133 did not have any informative tests. ^B^*N *= median number of informative families.

**Table 3:** Results for marker GGAA7D11 (chromosome 17) Results from FBAT analysis of the Framingham Heart Study offspring data using the unadjusted test for association between marker GGAA7D11 (chromosome 17) and high blood pressure and the adjusted test using regular smoking (>1 pack/day) as a covariate.

Allele		Unadjusted Test			Adjusted Test		
		
Name^A^	Frequency	No. Tests	% Significance	Median^B ^*N*	No. Tests	% Significance	Median^B ^*N*
262	0.099	100	0	26	100	46	63
266	0.175	100	0	45	100	0	94
270	0.091	100	0	35	100	0	70
274	0.135	100	87	40	100	89	86
278	0.206	100	0	48	100	0	103
282	0.157	100	0	43	100	0	90
286	0.083	100	35	31	100	6	59
290	0.099	100	0	13	100	0	25
262	00.032	100	0	26	100	46	63

^A^Alleles 254, 258, 283, and 294 did not have any informative tests. ^B^*N *= median number of informative families.
